# Effect of ret/PTC 1 rearrangement on transcription and post-transcriptional regulation in a papillary thyroid carcinoma model

**DOI:** 10.1186/1476-4598-5-70

**Published:** 2006-12-11

**Authors:** Susanne Cahill, Paul Smyth, Stephen P Finn, Karen Denning, Richard Flavin, Esther M O'Regan, Jinghuan Li, Astrid Potratz, Simone M Guenther, Richard Henfrey, John J O'Leary, Orla Sheils

**Affiliations:** 1 Dept. of Histopathology, University of Dublin, Trinity College, Dublin, Ireland; 2 Dept. of Pathology, Dublin Dental School and Hospital, Dublin, Ireland; 3 Applied Biosystems, Foster City, CA, USA

## Abstract

**Background:**

microRNAs (miRNAs) are a group of non-coding single stranded RNAs measuring approximately 22 nt in length that have been found to control cell growth, differentiation and apoptosis. miRNAs negatively regulate their target genes and recently have been implicated in tumourigenesis. Furthermore, miRNA expression profiling correlates with various cancers, with these genes thought to act as both tumour suppressors and oncogenes. ret/PTC 1 is an oncogene with constitutive kinase activity implicated in the development of papillary thyroid carcinoma (PTC). This rearrangement leads to aberrant MAPK activation that is implicated in PTC tumourigenesis.

**Aim:**

The aim of this study was to identify the effect that ret/PTC 1 has on transcription and post-transcriptional regulation in PTC by using DNA microarray and microRNA analysis.

**Results:**

DNA microarray analysis revealed a group of genes differentially expressed between normal thyroid cell lines and those harbouring a ret/PTC 1 rearrangement.

Furthermore, a unique miRNA expression signature differentiated between PTC cell lines with ret/PTC 1 and a normal thyroid cell line. 21 miRNAs showed significant overexpression and 14 miRNAs showed underexpression in these cell lines when compared to normal thyroid. Several of these up/down regulated miRNAs may be involved in PTC pathogenesis.

## Background

In 1985, *RET *was first identified as a novel oncogene. *RET *encodes a receptor tyrosine kinase, the ligands of which belong to the glial cell line-derived neurotropic factor (GDNF) family [1]. Chimeric *RET *oncogenes, designated ret/PTC, display a ligand independent constitutive tyrosine kinase activity and are implicated in the development of papillary thyroid carcinoma (PTC). They are formed from the juxtaposition of the genomic region coding for the tyrosine kinase domain of RET with the 5'-promoter regions of a variety of unrelated genes. At least 15 chimeric mRNAs involving ten distinct donor genes have been described. Of these, the most commonly occurring in PTC are ret/PTC 1 [H4-(CCDC6)-RET] [2] and ret/PTC 3 [ELE1-RET] [3].

Papillary thyroid carcinoma is the most frequently occurring type of thyroid malignancy accounting for approximately 80%–90% of cases. A unique feature of papillary thyroid cancers is that oncogenes such as ret/PTC, activate effectors that signal along the mitogen activated protein kinase (MAPK) pathway playing important roles in the pathogenesis of thyroid tumourogenesis. Genetic studies suggest that ret/PTC activation is one of the key first steps in thyroid cancer pathogenesis. The reported prevalence of ret/PTC rearrangements in papillary carcinoma varies from 3% to as high as 85% with the highest incidence in radiation associated PTC. This variation reflects the different sensitivities of the techniques used as well as geographic variations and the influence of environmental factors such as ionizing radiation exposure [4,5]. Several reports have shown that ret/PTC rearrangements are in general associated with classic papillary architecture in thyroid carcinoma and RET has been suggested to cause structural and nuclear peculiarities of PTC. PTCs expressing ret/PTC tend to be small and do not exhibit a tendency to progress to an aggressive phenotype [6-8]. However, the exact role of ret/PTC in the context of papillary thyroid carcinoma remains unclear and the complete repertoire of genes and signalling pathways involved in pathogenesis of thyroid disease remains poorly defined. A further shortcoming remains in the inadequacy of diagnostic and prognostic markers with frequent reliance on conventional morphological assessment.

Recently it has been proposed that microRNAs (miRNAs), small non coding RNAs, may play a role in tumour formation including PTC development. Ambrose et al described Lin-4 over a decade ago and there has been an explosion of interest in the area since [9,10]. microRNAs are cleaved from hairpin shaped precursors to yield mature miRNAs of approximately 22 nucleotides in length. miRNAs negatively regulate gene expression and function by targeting mRNAs for cleavage or translational repression depending on the degree of complementarity between the miRNA and the target mRNA [11,12]. Because of their relatively recent discovery, little is known about the exact processes in which miRNAs function or the exact nature of their regulation. Studies on the regulatory roles of miRNAs in a variety of organisms suggest that they have critical roles in central biological processes and are regulators of in development, cell proliferation, cell differentiation, stress resistance, metabolism and apoptosis, all of which are involved in tumourigenesis [13,14]. Not surprisingly as a consequence aberrant regulation of miRNA expression has been implicated in human disease including cancer. Furthermore, a recent study showed that greater than 50% of annotated human miRNAs are located in areas of the genome known as fragile sites that are associated with cancer [15]. This further supports a role for miRNAs in cancer progression. Differential expression of miRNAs has been shown between malignant and normal tissues of the same tissue type and between different types of tumour. This has lead to the hypothesis that tumours may each have a discrete miRNA signature [16-18]. It has also been reported that numerous oncogenes and tumour suppressor genes are potentially regulated by miRNAs, or indeed miRNAs may act as oncogenes or tumour suppressor genes themselves. miRNA species may therefore be potentially used as biomarkers for tumour diagnostics and treatment.

Several groups have used a variety of techniques in order to quantify miRNAs the most common including oligonucleotide microarray technology, bead based flow cytometric analysis, a modified invader assay and a new single molecule technique. Each of these methods has distinct advantages and disadvantages. Real-time PCR however, has unparalleled sensitivity and specificity. It is also more quantative than DNA microarray technology which is the preferred method in recent publications. The TaqMan^® ^microRNA RT-PCR method used in this study is a new real time quantification method to accurately detect mature miRNAs. The stem-loop structure of the primers is specific to the 3' end of the mature miRNA and creates a steric hindrance to prevent priming of the precursor miRNA. The aim of this study was to identify the effect the ret/PTC 1 oncogene has on transcription and post-transcriptional regulation in tumourigenesis by using a combination of DNA microarray and microRNA analysis.

## Results

### Differential gene expression using DNA Microarrays

#### Upregulated genes

In order to investigate differential gene expression in papillary thyroid carcinoma cell lines, a comparison was made between the two cell lines harbouring ret/PTC 1 rearrangement [Nthy-ori transfected with ret/PTC 1 and TPC-1] and normal thyroid cell lines. To identify potential markers of malignancy t-test with FDR correction (<0.1) were used to compare the two groups [19]. A p-value cut off of < 0.01 and a fold change difference of >2 resulted in a list of 47 genes (Table 1).

**Table 1:** Upregulated Genes in ret/PTC 1 cell lines v N-thy-ori

**GENE SYMBOL**	**GENE NAME**
**PLSCR4**	phospholipid scramblase 4
**Unassigned**	Unassigned
**Unassigned**	Unassigned
**TXNIP**	thioredoxin interacting protein
**IFI44**	interferon-induced protein 44
**PACS2**	phosphofurin acidic cluster sorting protein 2
**TBCIDI**	TBC1 domain family, member 1
**HLA-A**	major histocompatibility complex, class I, A
**PLSCR2**	phospholipid scramblase 2
**Unassigned**	Unassigned
**SBLF**	stoned B-like factor
**PCAF**	p300/CBP-associated factor
**C20orf128**	Chromosome 20 open reading frame 128
**MGC17403**	hypothetical protein MGC17403
**MCPH1**	microcephaly, primary autosomal recessive 1
**Unassigned**	Unassigned
**SH3BP1**	SH3-domain binding protein 1
**STXBP2**	syntaxin binding protein 2
**CAMLG**	calcium modulating ligand
**IFITM3**	interferon induced transmembrane protein 3 (1-8U)
**FRS3**	fibroblast growth factor receptor substrate 3
**SEMA3F**	sema domain, immunoglobulin domain (Ig), short basic domain, secreted, (semaphorin) 3F
**TRIM28**	tripartite motif-containing 28
**CYP2D6**	cytochrome P450, family 2, subfamily D, polypeptide 6
**HLA-C**	major histocompatibility complex, class I, C
**TMED6**	transmembrane emp24 protein transport domain containing 6
**CCNG1**	cyclin G1
**KLF10**	Kruppel-like factor 10
**RPL15**	Ribosomal protein L15
**PNPLA2**	patatin-like phospholipase domain containing 2
**CEBPB**	CCAAT/enhancer binding protein (C/EBP), beta
**HTRA1**	HtrA serine peptidase 1
**HLA-A**	major histocompatibility complex, class I, A
**ZNF329**	zinc finger protein 329
**C20orf29**	G protein-coupled receptor, family C, group 5, member C
**ARSA**	arylsulfatase A
**FAM100B**	family with sequence similarity 100, member
**C4orf14**	chromosome 4 open reading frame 14
**Membralin**	Membralin
**C1orf164**	Chromosome 1 open reading frame 164
**RPL32**	Ribosomal protein L32
**Unassigned**	Unassigned
**YHWAE**	tyrosine 3-monooxygenase/tryptophan 5-monooxygenase activation protein, epsilon polypeptide
**PAFAHIB**	platelet-activating factor acetylhydrolase, isoform Ib, alpha subunit 45 kDa
**GPRC5C**	G protein-coupled receptor, family C, group 5, member C
**C10orf104**	Chromosome 10 open reading frame 104

47 upregulated genes between ret/PTC 1 cell lines and normal.

#### Downregulated genes

**Figure 1:**
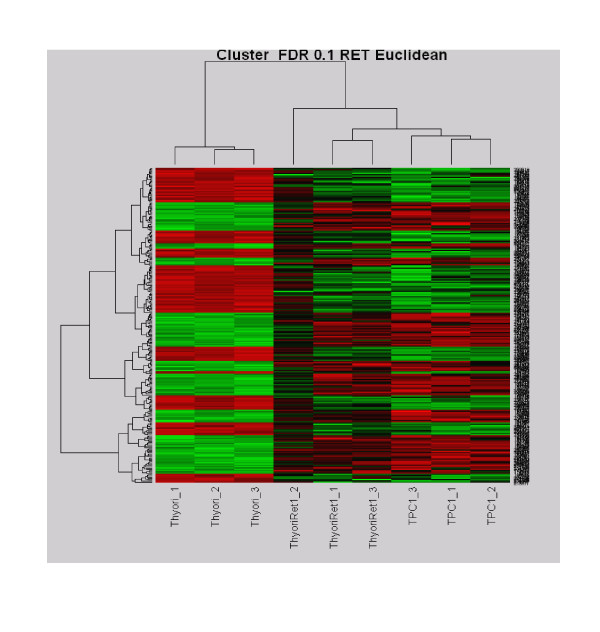
**Hierarchical clustering of ret/PTC 1 cell linesand N-thy-ori cell line based on the gene lists obtained from treatment comparison analysis**. The column dendrogram clearly shows cases clustering on the basis of RET rearrangement. To the left are the normal thyroid cell lines with no RET rearrangement and to the right are the cell lines with ret/PTC 1 rearrangement. Red denotes genes with relative increased expression and green denotes genes with relative decreased expression.

Similar cut off criteria were applied in analysing downregulated mRNA species. 29 genes were found to be statistically significant, see Table 2. Hierarchical clustering was performed based on statistically different genes (Fig. 1).

**Table 2:** Downregulated Genes in ret/PTC 1 cell lines Vs normal.

**GENE SYMBOL**	**GENE NAME**
**POMT2**	ring finger protein 126
**RNASEN**	ribonuclease III, nuclear
**PSMD2**	proteasome (prosome, macropain) 26S subunit, non-ATPase, 2
**CSRP1**	cysteine and glycine-rich protein 1
**DCTN5**	dynactin 5 (p25)
**ARL4A**	ADP-ribosylation factor-like 4A
**TXNRD1**	thioredoxin reductase 1
**Keratin type1**	Keratin Type1 cytoskeletal
**PSMC1**	proteasome 26S subunit, ATPase, 1
**TPM3**	tropomyosin 3
**BIT1**	Bcl-2 inhibitor of transcription
**TPM3**	Tropomyosin 1
**RAB32**	RAB32, member RAS oncogene family
**C16orf35**	Chromosome 16 open reading frame 35
**C21orf70**	Chromosome 21 open reading frame 70
**ITK**	IL2-inducible T-cell kinase
**TFRC**	transferrin receptor (p90, CD71)
**HISTIH4L**	histone 1, H4l
**TPM1**	tropomyosin 1 (alpha)
**Unassigned**	Unassigned
**KRT 18**	Keratin 18
**KIAA0256**	KIAA0256 gene product
**RNF126**	thioredoxin reductase 1
**FLJ37549**	proteasome (prosome, macropain) 26S subunit, non-ATPase, 2
**Unassigned**	unassigned;unassigned
**Unassigned**	protein-O-mannosyltransferase 2
**Unassigned**	unassigned;unassigned
**FGFBP1**	fibroblast growth factor binding protein 1
**NPM1**	nucleophosmin (nucleolar phosphoprotein B23, numatrin)
**Unassigned**	Unassigned

29 downregulated genes between the ret/PTC 1 cell lines and normal

### Differential miRNA expression using TaqMan^® ^microRNA assays

#### Upregulated

**Figure 2:**
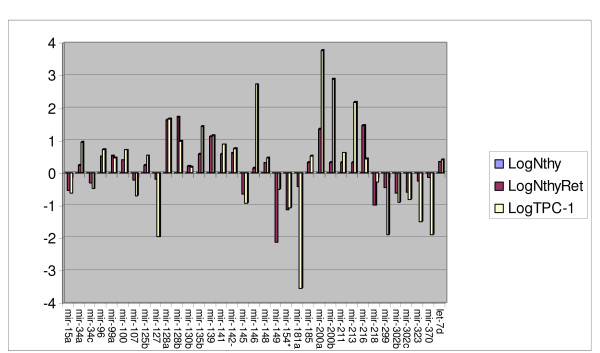
**Fold Change of miRNAs in ret/PTC harbouring cell lines Vs Normal**. Delta delta CT was performed using Nthy-ori 3-1 as a normal control. The log of the RQ values was used to plot the relative fold change of Nthy-Ret1 and TPC-1 against Nthy-ori 3-1. miRNAs are on the x axis.

**Figure 3:**
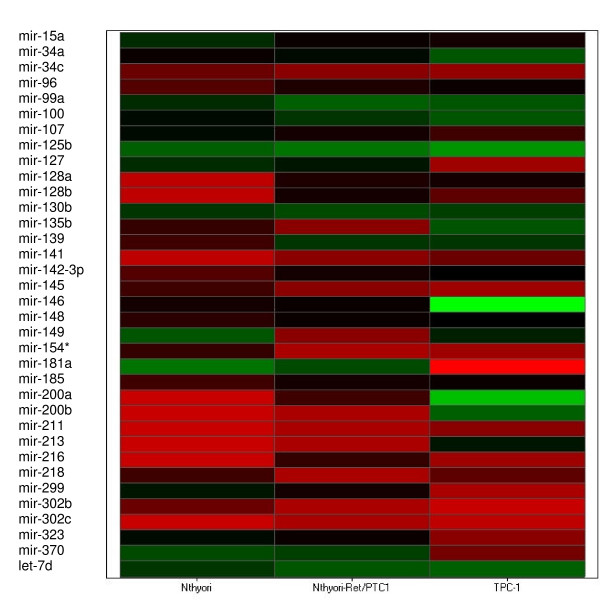
**Heat map of miRNA expression**. miRNA expression. Each miRNA listed was detected as significantly differentially expressed between ret/PTC 1 cell lines and N-thy-ori. The delta CT values for each miRNA were used to create the heat map.

miRNAs were considered to be significantly upregulated when there was a fold change of > 2 fold in PTC cell lines compared with the normal cell line N-thy-ori. miRNAs upregulated in both ret/PTC1 containing cell lines are listed in table 3, (21 in total). Four of these upregulated miRNAs showed significantly higher fold change than the other upregulated miRNAs, these are mir-128a [Fold Change (FC) = 42 (Nthyori-ret)/45 (TPC-1)], mir-128b [FC = 55/10], miR 139 [13/14] and mir-200a [25/5874]. (Table 3, Fig 2 and Fig 3).

**Table 3:** miRNA expression in ret/PTC 1 harbouring Cell lines Vs normal cell lines

**Upregulated**	**Downregulated**
mir-34a	mir-15a
mir-96	mir-34c
mir-99a	mir-107
mir-100	mir-127
mir-125b	mir-135b
mir-128a	mir-145
mir-128b	mir-149
mir-130b	mir-154*
mir-139	mir-181a
mir-141	mir-218
mir-142-3p	mir-299
mir-146	mir-302b
mir-148	mir-302c
mir-185	mir-323
mir-200a	mir-370
mir-200b	
mir-211	
mir-213	
mir-216	
Let-7d	

#### Downregulated

Similar criteria were used to determine miRNAs significantly under-expressed in ret/PTC 1 containing cell lines compared with N-thy-ori. A total of 14 miRNA species were identified in this cohort. Again four miRNAs showed significantly more underexpression compared to the other downregulated miRNAs. These miRNAs are mir- 154* [FC = 14/12], mir-181a [FC = 5/3401] and mir-302b [FC = 5/10] and mir302c [FC = 5/6]. (Table 3, Fig 2 and Fig 3)

### mRNA (transcription) and miRNA (regulation) expression correlation

miRNAs interact and regulate their target genes by cleavage or translational inhibition.

The current challenge is to identify biologically relevant targets that are regulated by individual miRNAs. Further complicating this task is the fact that every miRNA can potentially bind and regulate many mRNA targets and each mRNA can be bound and regulated by several miRNAs. miRNAs usually bind to their targets with incomplete complimentarity within the 3' untranslated region (UTR) of the mRNA target. Because of this the identification of targets is impossible with a simple BLAST search. Most bioinformatic methods use the first 2–8 bases of the mature miRNA sequence to search for complementarity to the 3' UTR of all expressed genes. This will yield a list of genes with diverse functions that a single miRNA can bind to. There are three publicly available databases for prediction of miRNA targets. These are miRBase [20], PICTAR [21], and TARGETSCAN [22]. For example, all animal miRNA sequences from the miRBase Sequence database are scanned against 3'-UTRs predicted from all available species in Ensembl along with *Caenorhabditis briggsae *and *Drosophila pseudoobscura*. P-values are assigned to individual miRNA-target binding sites, multiple sites in a single UTR, and sites that appear to be conserved in multiple species. The interface connects each miRNA to a list of predicted gene targets. Comparing the lists of the miRNA targets and differentially expressed genes, 15 differentially expressed genes from Table 1 and 2 were in the list of predicted targets for miRNAs from Table 3. This is illustrated in Table 5. Thus, there was correlation between the data sets obtained from the functional expression (microarray) and the regulatory (miRNA) data sets.

**Table 4:** Predicted targets for differentially expressed miRNAs

**miR-128a**	VEGFC, CDK9, RAB20, ST14, THRAP1, TXNIP, TRAF1, LGALs3, PTPN6, SLC6A7, ADAMTS6, ITGA3, RAB27A RAB34, ILI9, Elf3S4, GDF15, CASP8, CDKN2A, DUSPSP, HTRA 2, HLA-G, CDH 4, PCDH3, IFITM1, TBC1D2, SERPINA3 CASP10, ZNF432, NEK2, MAP3K1IPI, CDH24
**miR-128b**	PLK2, PRKD1, ADORA2B, ZNF385, CASC3, PAIP2, ITGB4BP CRKL, CDH24, ING5, BAG2, EGFR, ST14, THRAP1 CORO1CE2F, E2F3, PDGFRA, INSR, CITED2, STX16
**miR-139**	JUN, GAS2, MYST3, PTPRF, TGIF, XPO4, GDF10, MAP2, PCDH10
**miR-181a**	TMED4, TGFB1, PRKCD, MLK1, MAP3K10, WTAP, TSGA10 FOS, TUPBP1, VCAM, DROSHA, IRS2, TNFAIP1, RASSF1, ILISR1, DDIT4, PLAG1, GPBP1, PRR6, APIP, CKSIB IL2, FGFR2, RAP1B
**miR-370**	RAB3IP, PTPRG, CCN5, VSP6, CCL21, CNTNAP1, LTBP1 MAP3K8, GFRA2, MMP16, CCNK, ETV6, FGF7, THRAP2 BRCA1, FOSB
**miR-302c**	RAB43, CELSR3, NOTCH1, MET, CALCR, NOTCH2, THRAP2, TPO52, DAAMI, PDGFRA, MAD1A

Upregulated miRNAs in ret/PTC 1 cell lines = miR-128a, miR-128b, miR139

Downregulated miRNAs in ret/PTC 1 cell lines = miR-181a, miR-370, miR-302c

**Table 5:** miRNAs and Predicted Targets.

**Up-regulated Genes**	**Potential Binding Partner (down-regulated)**
IFI44	Mir-299
TXNIP	Mir-302c, mir-302b
CCNG1	Mir-181a
TRIM4	Mir-181a
HTRA1	Mir-302b, mir-302c
PNPLA2	Mir-181a, mir-34c
YWHAE	Mir-15a
CYP2D6	Mir-370
ZNF329	Mir-181a
**Down-regulated Genes**	**Potential Binding Partner (up-regulated)**
TPM1	Mir-181a, mir-185, mir-128b, mir-96, mir-128a
NPM1	Mir-200b
RAB32	Let-7d
FGFBP1	Mir-99a, mir-100
DROSHA	Mir-200b

## Discussion

The primary aim of this study was to try to elucidate the effects of the *RET *proto-oncogene on gene expression and transcriptional regulation in a matrix of cells lines harbouring ret/PTC 1 rearrangement. This was performed using Applied Biosystems whole genome expression array technology and TaqMan^® ^microRNA assays. The sequencing of the human genome and advances in microarray technology has significantly increased understanding of the molecular pathogenesis of thyroid tumour formation in recent years. On order to gain a better understanding of the genes and pathways involved in PTC and unveil potential biomarkers, several groups have applied DNA microarray technology to show the genetic profiles in PTC [23-26]. These profiles uncovered genes that were suggested to be involved in processes such cell adhesion and cell structure, inhibition and induction of apoptosis, cell proliferation and differentiation.

This study showed a group of differentially expressed genes in two cell lines contingent on a rearrangement of the *RET *oncogene when compared to normal thyroid. Among the genes found to be over-expressed, those involved in biological processes such as cell differentiation and proliferation were over-represented. Such genes included CEBPB, CCNG1, IFITM3 and HTRA1. CEBPB is a bZip transcription factor that cooperates with Stat 6 to activate transcription [27]. It has been shown to have a critical role in Ras mediated tumourigenesis and cell survival and has been implicated as a target for tumour inhibition. Studies have shown that it is expressed in the cytoplasm of PTC and is implicated to play a role in the regulation of thyroid specific genes and processes and has altered function in thyroid carcinoma [28]. It is involved in the positive regulation of genes involved in the immune and inflammatory response, a group which has been shown to be associated with ret/PTC 1 and tumour formation in this study and in previous thyroid literature [29]. CEBPB also has been shown to be a regulator of Cox-2; Cox-2 has recently been implicated as a potential biomarker in thyroid neoplasm [30].

HTRA1 [31] is a member of the trypsin family of serine proteases and is a regulator of cell growth. It has been implicated in the pathology of several diseases. Several papers have implicated matrix metalloproteases in PTC tumour progression; during tumour invasion they degrade protein components of the extracellular matrix aiding angiogenesis [32]. HTRA has been proven to cause the induction of matrix metalloproteases 1 and 3.

Cell signalling also showed an increase in ret/PTC 1 cell lines, SEMA3F is a protein involved in signalling. Endogenous SEMA3F causes cells to round and loose extracellular contacts a process which may contribute to the classic appearance of PTC. Membralin (C19orf6) is a novel gene originally cloned from a human ovarian cancer cell line. It is suggested to be a tumour associated marker in ovarian and colorectal cancer [33,34]. It was also shown to be over-expressed in ret/PTC 1 cell lines when compared to normal. It is interesting to hypothesize that there may be a link between membralin overexpression and the papillary architecture seen in PTC and in ovarian cancers.

Furthermore, genes involved in p53 pathway were overrepresented. P300/CBP- associated factor (PCAF) was one such gene and is a co- activator of the tumour suppressor p53. PCAF was found to be induced by p53 in breast cancer cell lines and indirectly regulated ERK1 effecting cellular proliferation [35].

Among the genes shown to be down-regulated in ret/PTC 1 cell lines when compared to normal, those involved in cell structure and motility were over-represented. Examples of such genes include DCTN5, TPM1, TPM3, CRP1 and Keratin type 1. Tumour cells are held together by direct cell – cell contact and by adhesion to the extracellular matrix. Loss of adhesion is thought to promote tumour invasiveness and increase the metastatic potential of carcinoma. Loss of these cell structure genes might also be involved in the papillary pattern of PTC. Tropomysoin (TM) has a role in regulating cellular functions associated with cytoskeletal remodelling. Suppression of TMs is a prominent feature of many transformed cells. TM1 expression is abolished in human breast cancer cell lines and also in the ret/PTC 1 cell lines in this study. This downregulation may destabilize microfilament architecture and facilitate survival of neoplastic cells and promote malignant growth [36]. PSMD2 (TRAP2) participates in TNF signalling pathway and was found to be downregulated. This suggested a role for PSMD2 in cell survival. Rab32 inactivation may represent a component of the oncogenic pathway of microsatellite-unstable gastrointestinal adenocarcinoma. It encodes an A-kinase anchoring protein and is often hypermethylated in gastric and colon cancers and was found to also be downregulated in PTC cell lines. Rab32 has also been shown to induce apoptosis [37,38].

Of particular interest in this study was the discovery of the underexpression of DROSHA in PTC cell lines. This is the core nuclease that executes the initiation step of miRNA processing in the nucleus. DROSHA collaborates with Dicer in stepwise processing of miRNAs and has a key role in miRNA mediated gene regulation processes such as development and differentiation [39]. Components of the miRNA-machinery have been implicated in tumourigenesis and reduced levels have been shown to be associated with cancer [40]. Its differential expression may effect the expression of certain miRNAs such as those in table 3 and consequently play a role in gene expression regulation.

This data corroborates the importance of these pathways in PTC. The genes involved may play a role in tumour initiation and in the morphological characteristic of classic PTC due to the ret/PTC 1 oncogene. Several reports have shown that ret/PTC 1 rearrangements are in general associated with classic papillary architecture in thyroid carcinoma. The profiles examined contained genes that were suggested to be involved in processes such as cell adhesion and cell structure, such as tropomysoins, and inhibition and induction of apoptosis. This aberrant expression has an impact on the molecular structure and the extracellular matrix which may facilitate destabilization of the microfilament architecture. While also adding to tumour spread this phenomenon may contribute to the classic papillary appearance associated with this rearrangement.

Gene expression has recently been shown to be regulated by small noncoding RNAs called microRNAs (miRNAs). It is believed that miRNAs are responsible for fine tuning gene expression and are thought to regulate approximately 30% of the human genome, making them one of the largest classes of gene regulator. Several clues have pointed to the potential role of miRNAs in cancer and suggest that aberrations in miRNA expression may be important in tumour progression by regulating genes and pathways involved in cancer associated processes. Studies have reported that reduced levels of the Let-7 family of miRNAs have an association with lung cancer tumourigenesis [41,42]. The miRNA cluster of miR-17- miR-92 is highly expressed in many B-cell chronic lymphomas [43]. Furthermore, Mir 15a-Mir 16 expression is upregulated in B-cell chronic lymphoma and leukaemia and falls within a region thought to harbour a tumour suppressor gene. Glioblastomas and breast tumours have also been reported to show over expression of miR-21 [44,45].

This study examined, by real-time PCR, the expression of miRNA in papillary thyroid carcinoma cell lines with ret/PTC1 rearrangement.

Tables 3 shows a set of differentially expressed miRNAs in 2 cell lines with ret/PTC1 rearrangement when compared to normal thyroid cell line. 21 miRNAs were found to be overexpressed and 14 miRNAs were underexpressed in both cell lines when compared to Nthy-ori 3-1. These miRNAs are effected by direct ret/PTC 1 expression or by the expression of other genes that are induced by ret/PTC 1 such as transcription factors. Little is known about the function of these miRNAs and only a subset has been found to be differentially expressed in other cancers. Of the miRNAs in table 3, miR-181a expression was down-regulated and expression of miR-200b and miR-141 was up-regulated. Ciafre et al found miR-181a expression to be downregulated in glioblastoma [45]. Studies have reported that miR-181 upregulated during differentiation and suggest that miR-181 downregulates the homeobox protein Hox-A11 (a repressor of the differentiation process), thus establishing a functional link between miR-181 and the complex process of differentiation. In fully differentiated tissue miR-181 was found to be downregulated, PTCs are well-differentiated carcinomas therefore this may explain downregulation of miR-181a in this study.

miR-200b and miR-141 have also been shown to be highly overexpressed in malignant cholangiocytes and in colon carcinoma [46,47]. Studies showed that their inhibition increased sensitivity to the anticancer drug gemcitabine and also decreased cell growth suggesting a role for their involvement in tumour formation.

An immediate obstacle facing research groups at present is the lack of confirmed miRNA gene targets. Although multiple approaches have been suggested from bioinformatics including many prediction algorithms accurate target prediction and validation are still major obstacles facing miRNA researchers. Each miRNA found to be significantly up/down regulated from table 3 was imputed into the algorithms, miRBase, PICTAR and TARGETSCAN to search for potential miRNA:miRNA target pairings. The potential binding partners of 6 of these miRNAs are listed in table 4. It is interesting to note that these differentially expressed miRNAs potentially bind and regulate a number of genes that have implications in thyroid carcinoma progression such as genes involved in thyroid function, MAPKKK cascade, retinoic acid receptors, cell adhesion and cell structure associated genes, genes involved in cell signalling, G-protein mediated signalling, oncogenesis and cell cycle control. Examples of these genes include thyroid hormone interacting protein, protocadherin, MAP3K10, KIT, PDGFRA and exportin 4. Therefore, it seems plausible that this subset of miRNAs may be important in the progression of thyroid carcinoma by regulating genes involved in thyroid neoplasia such as those found in this study discussed above.

Analysis of DNA microarray data showed a group of genes that are differentially expressed in ret/PTC1 harbouring cell lines (Table 1 and 2). Comparing this list of genes with the list of putative miRNA targets yielded from analysis using PICTAR, TARGETSCAN and miRBase showed that 15 of the differentially expressed genes are targeted by several miRNAs that are significantly up/downregulated in cell lines with ret/PTC 1 rearrangement. This data is shown in Table 5. Again it is interesting to speculate that these genes are regulated by their corresponding miRNA thereby contributing to tumour development in thyroid. It remains difficult to identify the precise target mRNA of each miRNA species. Therefore without further experimental investigation any mRNA:miRNA partners are essentially speculative and selection involves a degree of subjectivity.

Recent studies on miRNA deregulation in PTC found an aberrant miRNA expression profile in PTCs compared to normal thyroid tissues. In particular, a significant increase in miR- 222, miR-221, miR-146 and miR-181b was seen [48,49]. In this study miR-146 was upregulated 500 fold in TPC-1 cell line only. It is possible this miRNA may play a role in a late stages of tumour formation and progression; given the transfected cell line had only been exposed to ret/PTC1 for a short period (3 passages). Alternatively, the difference may reflect the accumulation of several genetic insults. The other miRNAs [miR-222, miR- 221, miR-181b], however, were not found to be upregulated significantly. This may be due to the differences in experimental approaches; de la Chapelle et al and Fusco A et al used miRNA microarrays. They also used different chemistry and fresh tissue samples; also the *RET *status of each PTC case was not disclosed.

This field of miRNA research is young and many important questions still remain. It is clear that miRNAs exert a regulatory role on protein coding genes in a variety of species and their deregulation may contribute to the development of human disease. However, the precise biological function of miRNAs has yet to be fully elucidated.

Even without more in-depth knowledge of their exact function or role in PTC, it seems reasonable that a distinct signature of miRNAs or miRNA expression profiles assigned for thyroid tumours might be used as an adjunct in diagnosis and prognostication of thyroid neoplasia. It has been suggested that miRNA signatures may be more effective than profiles of protein coding genes in distinguishing tumours from benign lesions. Fortunately miRNAs, unlike mRNA targets remain intact in routinely collected paraffin embedded samples, a fact which increases their potential use as clinical biomarkers. miRNAs also have the potential as possible treatment targets. One example of potential miRNA targeted therapy has been shown with miR-127 in bladder cancer. This suggests the possibility of direct targeting of miRNAs that are amplified or upregulated in patient tumours.

## Conclusion

This study is an *in vitro *model for the biological and regulatory processes that occur in human thyroid diseases due to ret/PTC1 rearrangement. As miRNAs are stable, abundant and easily detectable they represent ideal candidates for effective diagnostic biomarkers. Future exploration on a larger cohort of samples will hopefully determine the correlation between these miRNAs and their host genes in PTC and help aid in the identification of miRNA biomarkers.

## Methods

### Cell culture and transfection

Nthy-ori 3-1 (ECACC, Wiltshire, UK) is a normal thyroid follicular epithelial cell line.

The cell line is derived from normal thyroid tissue of an adult that has been transfected with a plasmid encoding for the SV40 large T gene.

TPC-1 is a cell line derived from a papillary thyroid carcinoma. This cell line expresses the ret/PTC-1 oncogene.

Both cell lines were grown to confluence in a humidified atmosphere containing 5% CO2 at 37°C in the following plating medium: RPMI 1640 with 2 mM L-glutamine, 10% Foetal calf serum (FCS), Penicillin (100 U/ml) and Streptomycin (100 μg/ml). ret/PTC 1 transcript was cloned into the multiple cloning site of pcDNA4/TO using standard molecular techniques involving restriction and ligation. This is part of the TREx tetracycline-regulated mammalian expression system (Invitrogen, UK). Expression of the gene of interest from pcDNA4/TO is controlled by the CMV promoter (into which 2 copies of the tet operator 2 (TetO2) sequence have been inserted in tandem.) Each TetO2 sequence serves as a binding site for 2 molecules of the Tet repressor. The second major component of the T-REx system is the pcDNA6/TR^© ^regulatory vector which expresses high levels of the TetR gene under the control of the human CMV promoter. In the absence of tetracycline, the Tet repressor forms a homodimer that binds with high affinity to each TetO2 sequence in the promoter of the inducible expression vector. Upon addition, tetracycline binds with high affinity to each Tet repressor homodimer and causes a conformational change in the repressor that renders it unable to bind to the Tet operator. The Tet repressor:tetracycline complex then dissociates from the Tet operator and allows induction of transcription from the gene of interest. pcDNA6/TR and pcDNA4/TO-ret/ptc1 were tranfected into N-thy-ori cells using Genejuice™ transfection agent (Novagen, Germany) using the recommended protocol and grown in the presence selection agents, Blasticidin (Sigma Aldrich) and Zeocin (Sigma Aldrich) to yield pure cultures.

### Nucleic acid extraction

Following trypsinisation of cultured cells, cell pellets (3–4 × 106 cells) were collected and RNA was extracted using RNeasy^® ^mini kit (Qiagen Ltd., West Sussex, UK). See Figure 1. RNA quantity and quality are assessed using the NanoDrop^® ^ND-1000 Spectrophotometer (Wilmington, USA) and the RNA 6000 Nano LabChip^® ^Kit in conjunction with the Agilent 2100 Bioanalyser (Agilent technologies, Waldbronn, Germany). Taqman^® ^RT-PCR was used for Ret/PTC1 rearrangement detection. Primers and probes used in this experiment were designed and used according to the Applied Biosystems (Foster City, CA, USA) Assays-by-Design SM service. Amplification and analysis was performed on an ABI Prism 7000 Sequence Detection System (Applied Biosystems, CA, USA) (48°C for 30 min then 92°C for 15 sec, 60°C for 1 min × 40 cycles).

### Microarray analysis

The Applied Biosystems 1700 Expression Array System is based on a microarray design that represents the whole human genome. The V.2 array has 32,878 60-mer oligonucleotide probes for the interrogation of 29,098 individual human genes and more than 1,000 control probes. V2 arrays were used to analyse the transcriptional profiles of the cell line RNA samples in this study. Digoxigenin-UTP labeled cRNA was generated and linearly amplified from 2 μg of total RNA using Applied Biosystems Chemiluminescent RT-IVT Labelling Kit v 2.0 following the manufacturer's protocol. Array hybridization, chemiluminescence detection, image acquisition and analysis were performed using Applied Biosystems Chemiluminescence Detection Kit and 1700 Chemiluminescent Mircoarray Analyzer following manufacturer's guidelines. Each microarray was initially pre-hybridised at 55°C for 1 hr in hybridization buffer with blocking reagent. 10 μg of labeled cRNA targets were fragemented by incubating with fragmentation buffer at 60°C for 30 min, mixed with internal control target (ICT, 24-mer oligo labeled with LIZ fluorescent dye) and hybridized to each pre-hybed microarray in a 1.5 ml volume at 55°C for 16 hr. After hybridization, the arrays were washed with hybridization wash buffer and chemiluminscence rinse buffer. Enhanced chemiluminescenct signals were generated by incubating arrays with anti-digoxigenin alkaline phosphatase, enhanced with Chemiluminescence Enhancing Solution and finally by adding Chemiluminescence Substrate. Images were collected for each microarray using the 1700 analyser. Images were autogridded and the chemiluminescent signals were quantified, corrected for background and spot and spatially normalized. Replicates were performed.

### Statistical analysis

Microarrays were analysed using R version 1.9.1 [a free language and environment for statistical analysis and graphics] (R Development Core Team, 2004). Arrays were normalised using Quantile normalisation. The primary means of identifying genes that were differentially expressed between groups was based on fold change and statistical significance. An ANOVA test was used to generate p-values for statistical difference between groups. P-values were then adjusted for multiple comparisons using a technique described by Benjamini and Hochberg with a cut of FDR <0.1 [19]. Genes were deemed statistically different between groups fold change >2 fold. Hierarchical clustering was performed based on statistically different genes (Fig. 1). Gene ontology analysis was performed using an online database known as PANTHER (panther.appliedbiosystems.com). Its functions include the ability to merge and differentiate genes within lists and the ability to check for over-representation of genes with a particular biological function, or involved in a specific biological pathway.

### miRNA analysis

Applied Biosystems TaqMan^® ^microRNA (miRNA) assays are designed to detect and quantify mature miRNAs using a looped-primer real time PCR. The human early access panel used in this study contained 160 individual assays covering many of the identified human miRNAs. The assay involved 2 steps: Step one; a Stem-looped RT, and Step two; a Real Time PCR. Briefly, single stranded cDNA was generated from total RNA sample by reverse transcription using the Applied Biosystems High-Capacity cDNA Archive Kit (Applied Biosystems, CA, USA) following manufacturer's protocol. RT reactions contained 10 ng of total RNA, 50 nM stem-looped RT primer, 1 × RT buffer, 0.25 mM each of dNTPs, 3.33 U/Ol Multiscribe reverse transcriptase and 0.25 U/Ol RNase Inhibitor. PCR amplification was carried out using sequence specific primers on the Applied Biosystems 7900 HT Fast Real-Time PCR system. The reactions were incubated in a 96-well optical plate at 95°C for 10 min, following by 40 cycles of 95°C for 15s and 60°C for 10 min. Analysis of relative miRNA expression data was performed using ΔΔCT method with hsa-let-7a as an endogenous control. Two negative controls were also used, ath-mir159a and cel-lin-4.

## Competing interests

The author(s) declare that they have no competing interests.

## Authors' contributions

SC performed the microarray and miRNA analysis and wrote original and final versions of the manuscript. PS, EOR, SF carried out statistical and analytical analysis of microarray data and helped draft the manuscript. JH, KD carried out cell culture. AP, SG, RH helped with the analysis of the miRNA data. JOL and OS conceived the study and helped write the original and final versions of this manuscript.

## References

[B1] Takahashi M, Ritz J, Cooper GM (1985). Activation of a novel human transforming gene, ret, by DNA rearrangement. Cell.

[B2] Smanik PA, Furminger TL, Mazzaferri EL, Jhiang SM (1995). Breakpoint characterization of the ret/PTC oncogene in human papillary thyroid carcinoma. Hum Mol Genet.

[B3] Santoro M, Dathan NA, Berlingieri MT, Bongarzone I, Paulin C, Grieco M, Pierotti MA, Vecchio G, Fusco A (1994). Molecular characterization of RET/PTC3; a novel rearranged version of the RET proto-oncogene in a human thyroid papillary carcinoma. Oncogene.

[B4] Sheils O (2005). Molecular classification and biomarker discovery in papillary thyroic carcinoma. Expert Rev Mol Diagn.

[B5] Kondo T, Ezzat S, Asa SL (2006). Pathogenetic mechanisms in thyroid follicular-cell neoplasia. Nat Rev Cancer.

[B6] Fischer AndrewH, Bond JaneA, Panya Taysavang, Eugene O, David Battles, Thomas Wynford (1998). Papillary Thyroid Carcinoma oncogene (RET/PTC) alters the nuclear envelope and chromatin structure. Am J Pathol.

[B7] Smyth P, Finn S, Cahill S, O'Regan EM, Flavin R, O'Leary JJ, Sheils O (2005). Ret/PTC and BRAF act as distinct molecular, time dependent triggers in a sporadic Irish cohort of papillary thyroid carcinoma. Int J Surg Pathol.

[B8] Giovanni Tallini, Massimo santoro, Mary Helie, Francesca Carlomagno, Giuliana Salvatore, Gennaro Chiappetta, Luisa Carcangiu Maria, Alfredo Fusco (1998). RET/PTC oncogene activation defines a subset of papillary thyroid carcinomas lacking evidence of progression to poorly differentiated or undifferentiated tumour phenotypes. Clin Cancer Res.

[B9] Lee RC, Feinbaum RL, Ambrose V (1993). The *C-elegans *heterochronic gene *lin-4 *encodes small RNAs with antisense complementarity to *lin-14*. Cell.

[B10] Feinbaum R, Ambrose V (1999). The Timing of *lin-4 *RNAaccumulation controls the timing of postembryonic developmental events in *Caenorhabditis elegans*. Dev Biol.

[B11] Esquela-Kerscher A, Slack FJ (2006). Oncomirs-MicroRNAs with a role in cancer. Nat Rev Cancer.

[B12] Sevignani C, Calin GA, Siracusa LD, Croce CM (2006). Mammalian MicroRNAs: a small world for fine-tuning gene expression. Mamm Genome.

[B13] Miska EA (2005). How MicroRNAs control cell division, differentiation and death. Curr Opin Genet Dev.

[B14] Hwang HW, Mendell JT (2006). MicroRNAs in cell proliferation, cell death and tumourigensis. British J of Cancer.

[B15] Calin GA, Sevignani C, Dumitru CD, Hyslop T, Noch E, Yendamuri S, Shimizu M, Rattan S, Bullrich F, Negrini M, Croce CM (2004). Human microRNA genes are frequently located at fragile sites and genomic regions involved in cancers. PNAS.

[B16] Lu J, Miska EA, Alvarez-Saavedra E, Lamb J, Peck D, Sweet-Cordero A, Ebert BL, Mak RH, Ferrando AA, Downing JR, Jacks T, Horvitz R, Golub TR (2005). MicroRNA expression profiles classify human cancers. Nature.

[B17] Volinia Stefano, Calin GeorgeA, Liu Chang-Gong, Ambs Stefan, Cimmino Amelia, Petrocca Fabio, Visone Rosa, Iorio Marilena, Roldo Claudia, Ferracin Manuela, Prueitt RobynL, Yanaihara Nozumu, Lanza Giovanni, Scarpa Aldo, Vecchione Andrea, Negrini Massimo, Harris CurtisC, Croce CarloM (2006). A microRNA expression signature of human solid tumours defines cancer gene targets. PNAS.

[B18] Liu Chang-Gong, Calin George Adrian, Meloon Brian, Gamliel Nir, Sevignani Cinzia, Ferracin Manuela, Dumitru Calin Dan, Shimizu Masayoshi, Zupo Simona, Dono Mariella, Alder Hansjuerg, Bullrich Florencia, Negrini Massimo, Carlo CroceM (2004). An oligonucleotide microchip for genome wide microRNA profiling in human and mouse tissues. PNAS.

[B19] Hochberg Y, Benjamini Y (1990). More powerful procesdures for multiple significance testing. Stat Med.

[B20] Griffiths-Jones Sam, Grocock RussellJ, van Dongen Stijn, Bateman Alex, Enright AntonJ (2006). miRBase: microRNA sequences, targets and gene nomenclature. Nucleic Acids Res.

[B21] Krek A, Grun D, Poy M, Wolf R, Rosenburg L, Epstein EJ, MacMenamin P, da Piedade I, Grunsalus K, Stoffel M, Rajewsky N (2005). Combinatorial MicroRNA Target Predictions. Nat Genet.

[B22] Lewis BP, Shih I, Jones-Rhoades MW, Bartel DP, Burge CB (2003). Prediction of Mammalian MicroRNA Targets. Cell.

[B23] Huang Y, Prasad M, Lemon WJ, Hampel H, Wright FA, Kornacker K, LiVolsi V, Frankel W, Kloos RT, Eng C, Pellagata NS, De la Chapella (2001). Gene expression in papillary thyroid carcinoma reveals highly consistent profiles. PNAS.

[B24] Yano Y, Uematsu N, Yashiro T, Hara H, Ueno E, Miwa M, Tsujimoto G, Aijoshi Y, Uchida K (2004). Gene expression profiling identifies platelet-derived growth factor as a diagnostic molecular marker for papillary thyroid carcinoma. Clin Cancer Res.

[B25] Finley DJ, Arora N, Zhu B, Gallagher L, Fahey TJ (2004). Molecular profiling distinguishes papillary carcinoma from benign thyroid nodules. J Clin Endocrinol Metab.

[B26] Jarzab B, Wiench M, Fujarewicz K, Simek K, Jarzab M, Oczko-Wojciechowska M, Wloch J, Czarniecka A, Chmielik E, Lange D, Pawlaczek A, Szpak S, Gubala E, Swierniak A (2005). Gene Expression Profile of Papillary Thyroid Cancer: Sources of Variability and Diagnostic Implications. Cancer Res.

[B27] Mikita T, Kurama M, Schindler U (1998). Synergistic activation of the germline epsilon promoter mediated by Stat6 and C/EBP beta. J Immunol.

[B28] Pomerance M, Mockey M, Young J, Quillard J, Blondeau JP (2006). Expression, hormonal regulation and subcellular localization of CCAAT/enhancer-binding protein-beta in rat and human thyrocytes. Thyroid.

[B29] Borrello MG, Alberti L, Fischer A, Degl'Innocenti D, Ferrario C, Gariboldi M, Marchesi F, Allavena P, Greco A, Collini P, Pilotti S, Cassinelli G, Bressan P, Fugazzola L, Mantovani A, Pierotti MA (2005). Induction of a proinflammatory program in normal human thyrocytes by the ret/PTC1 oncogene. PNAS.

[B30] Specht MC, Tucker ON, Hocever M, Gonzalez D, Teng L, Fahey TJ (2002). Cyclooxygenase-2 expression in thyroid nodules. J Clin Endocrinol Metab.

[B31] Grau S, Richards PJ, Kerr B, Hughes C, Caterson B, Williams AS, Junker U, Jones SA, Clausen T, Ehrmann M (2006). The role of human HTRA1 in arthric disease. J Biol Chem.

[B32] Wasenius VM, Hemmer S, Kettunen E, Knuutila S, Franssile K, Joensuu H (2003). Hepatocyte Growth factor receptor, matrix metalloproteinase-11, tissue inhibitor of metalloproteinase-1 and fibronectin are up-regulated in papillary thyroid carcinoma: a cDNA and tissue microarray study. Clin Cancer Res.

[B33] Andersson O, Von Euler G (2002). Characterization and expression of the gene encoding membralin, an evolutionary conserved protein expressed in the central nervous system. Brain Res Gene Expr Patterns.

[B34] Chen YC, Davidson B, cheng CC, Maitra A, Giuntoli RL, Hruban RH, Wang TL, Shih IM (2005). Identification and characterisation of membralin a novel tumour associated gene in ovarian carcinoma. Biochem Biophys Acta.

[B35] Chu B, Tran K, Ku T, Crowe DL (2005). Regulation of ERK1 gene expression by coactivator proteins. Biochem J.

[B36] Raval GN, Bharadwaj S, Levine EA, Willingham MC, Geary RL, Kute T, Prasad GL (2003). Loss of expression of tropomyosin-1, a novel class 11 tumour suppressor that induces anoikis in primary breast tumours. Oncogene.

[B37] Alto NM, Soderling J, Scott JD (2002). Rab 32 is an A-kinase anchoring protein and participates in mitochondrial dynamics. J Cell Biol.

[B38] Shibata D, Mori Y, Cai K, Zang L, Yin J, Elahi A, Hamelin R, Wong YF, Lo WK, Chung TK, Sato F, Karpeh MS, Meltzer SJ (2006). Rab 32 hypermethylation and microsatellite instability in gastric and endometrial adenocarcinoma. Int J Cancer.

[B39] Lee Y, Ahn C, Han J, Choi H, Kim J, Yim J, Lee J, Provost P, Radmark O, Kim S, Kim VN (2003). The nuclear RNase 111 Drosha initiates microRNA processing. Nature.

[B40] Irvin-Wilson CV, Chauduri G (2005). Alternative inititiation and splicing in dicer gene expression in human breast cells. Breast Cancer Res.

[B41] Yanaihara Nozomu, Caplen Natasha, Bowman Elise, Seike Masahiro, Kumamoto Kensuke, Yi Ming, Stephens RobertY, Okamoto Aikou, Yokota Jun, Tanaka Tadao, Calin George Adrian, Liu Chang-Gong, Croce CarloM, Harris CurtisC (2006). Unique microRNA molecular profiles in lung cancer diagnosis and prognosis. Cancer Cell.

[B42] Takamizawa J, Konishi H, Yanagisawa K, Tomida S, Osada H, Endoh H, Harano T, Yatabe Y, Nagino M, Nimura Y, Mitsudomi T, Takahashi T (2004). Reduced expression of the let-7 MicroRNAs in human lung cancers in association with shortened postoperative survival. Cancer Res.

[B43] Calin GA, Liu CG, Sevignani C, Ferracin M, Felli N, Dumitru CD, Shimizu M, Cimmino A, Zupo S, Dono M, Dell'Aquila ML, Alder H, Rassenti L, Kipps TJ, bullrich F, Negrini M, Croce CM (2004). MicroRNA profiling reveals distinct signatures in B cell chronic lymophocytic leukemias. PNAS.

[B44] Iorio MV, Ferracin M, Liu CG, Veronese A, Spizzo R, Sabbioni S, Magri E, Pedriali M, Fabbri M, Campiglio M, Menard S, Palazzo JP, Rosenberg A, Musiani P, Volinia S, Nenci I, Calin GA, Querzoli P, Negrini M, Croce CM (2005). MicroRNA gene expression deregulation in human breast cancer. Cancer Res.

[B45] Ciafre SA, Galardi S, Mangiola A, Ferracin M, Liu CG, Sabatino G, Maira G, Croce CM, Farace MG (2005). Extensive modulation of a set of micrornas in primary glioblastoma. Biochem Biophys Res Commun.

[B46] Bandres E, Cubedo E, Agirre X, Malumbres R, Zarate R, Ramirez N, Abajo A, Navarro A, Moreno I, Monzo M, Garcia-Foncillas J (2006). Identification by real time PCR of 13 mature mirnas differentially expressed in colorectal cancer and nontumoural tissues. Mol Cancer.

[B47] Guimares-Sternburg C, Meerson A, Shaked I, Soreq H (2006). Micro Modulation of megakaryoblast fate involves cholinergic signalling. Leuk Res.

[B48] Pallante P, Viscone R, Ferracin M, Ferraro A, Berlingieri MT, Troncone G, Chiappette G, Liu CG, Santoro M, Negrini M, Croce CM, Fusco A (2006). MicroRNA deregulation in human papillary thyroid carcinomas. Endocr Relat Cancer.

[B49] Huiling H, Jazdzewski K, Li W, Liyanarachchi S, Nagy R, Volinia S, Calin GA, Liu CG, Franssila K, Suster S, Kloos RT, Croce CM, De la Chapelle A (2005). The role of microRNA genes in papillary thyroid carcinoma. PNAS.

